# Spectroscopic stimulated Raman scattering imaging of highly dynamic specimens through matrix completion

**DOI:** 10.1038/lsa.2017.179

**Published:** 2018-05-04

**Authors:** Haonan Lin, Chien-Sheng Liao, Pu Wang, Nan Kong, Ji-Xin Cheng

**Affiliations:** 1 Department of Biomedical Engineering, Boston University, Boston, MA 02215, USA; 2 Department of Electrical & Computer Engineering, Boston University, Boston, MA 02215, USA; 3 Vibronix, Inc., West Lafayette, IN 47907, USA; 4 Weldon School of Biomedical Engineering, Purdue University, West Lafayette, IN 47907, USA

**Keywords:** optical microscopy, Raman scattering, vibrational spectroscopy

## Abstract

Spectroscopic stimulated Raman scattering (SRS) imaging generates chemical maps of intrinsic molecules, with no need for prior knowledge. Despite great advances in instrumentation, the acquisition speed for a spectroscopic SRS image stack is fundamentally bounded by the pixel integration time. In this work, we report three-dimensional sparsely sampled spectroscopic SRS imaging that measures ~20% of pixels throughout the stack. In conjunction with related work in low-rank matrix completion (e.g., the Netflix Prize), we develop a regularized non-negative matrix factorization algorithm to decompose the sub-sampled image stack into spectral signatures and concentration maps. This design enables an acquisition speed of 0.8 s per image stack, with 50 frames in the spectral domain and 40,000 pixels in the spatial domain, which is faster than the conventional raster laser-scanning scheme by one order of magnitude. Such speed allows real-time metabolic imaging of living fungi suspended in a growth medium while effectively maintaining the spatial and spectral resolutions. This work is expected to promote broad application of matrix completion in spectroscopic laser-scanning imaging.

## Introduction

Coherent Raman scattering imaging^1, 2, 3^, including stimulated Raman scattering (SRS) microscopy^4, 5, 6, 7, 8^ and coherent anti-Stokes Raman scattering (CARS) microscopy^9, 10, 11, 12^, is a burgeoning non-linear optical imaging technique that enables visualization of molecules in cells, tissues and functional materials. In SRS microscopy, two laser pulses, one at Stokes frequency (*ω*_s_) and the other at pump frequency (*ω*_p_), are tightly focused at the sample to generate images in a scanning manner. Speed and chemical specificity are two most important metrics in evaluation of an SRS imaging system. By focusing the beam energy at a single vibration mode, SRS has reached a speed of up to video rate^13^, opening doors for a plethora of applications including (but not limited to) observing drug delivery pathways to better engineer target-specific medicine^14^ and supplying contrast of cancer tissues for real-time histopathological analysis^15^. To improve chemical specificity, spectroscopic SRS imaging has been developed by either successively stacking a series of SRS images at different Raman shifts (frame by frame)^16, 17, 18, 19, 20, 21, 22, 23^ or recording a complete spectrum at each spatial location (multiplex)^24, 25, 26, 27, 28^. The extensive spectral information offered by spectroscopic SRS makes it possible to resolve molecules through overlapped Raman bands. Nevertheless, such improvement comes at the price of reduced imaging speed. Several previous designs suffer from a long adjustment time for frequency tuning due to the use of a translational stage, resulting in an acquisition time of several minutes^16, 17, 18, 21^. In the most recent frame-by-frame designs, frequency tuning via control of the angle of a scanning mirror was reported and reached an acquisition speed of a few seconds per stack^19, 20, 22^. Similar performances were also reported for multiplexed designs^24, 25, 26^ in which a spectrum was recorded within several microseconds at each spatial location. Currently, the speed of SRS imaging has reached video rate with four spectral channels^29^. However, further extending the speed limit while maintaining a sufficient number of spectral frames is a challenging task because each spectroscopic image stack requires tens to hundreds of times more pixel measurements than a single-color image. Increasing the speed by reducing the pixel dwell time is expected to significantly downgrade the sensitivity of the system because the SRS signal intensity decreases and is eventually overwhelmed by shot noise and thermal noise.

Several computational methods have been proposed to break the speed limit of spontaneous Raman imaging bounded by signal integration time. With use of the sparsity of natural images, increasing speed via compressed sensing has been reported to record a Raman image from a few random projections of the original data. Random projection measurements were experimentally implemented by either coding in the spectral domain using a spatial light modulator^30^ or collectively in the spatial and spectral domains using a digital micromirror device^31^ to modulate wide-field illuminated Raman images.

For spectroscopic imaging, the number of major spectral components is usually much smaller than the number of spectral frames, or in other words, a spectroscopic image can be treated as a low-rank matrix. The low-rank property implies significant information redundancy, and theories have shown that, for such a low-rank unknown matrix, one can accurately reconstruct the data from only a set of randomly sampled entries^32, 33^. The problem, known as matrix completion, arises in a plethora of areas, including collaborative filtering^34^, radio signal analysis^35^ and global positioning in sensor networks^36^, among others. By harnessing the idea of matrix completion, we demonstrate an effective method of improving the speed without harming the pixel dwell time by randomly sampling a small portion of pixels throughout the spectroscopic image stack. A regularized spectral unmixing (i.e., matrix factorization) algorithm is used to decompose the sparse spectroscopic image into spectral signatures and concentration maps. This algorithm is selected because the two output matrices can be used in direct visualization, and the reduced dimensions of the variables (due to the low rank of the spectroscopic image) make it faster than algorithms focusing on the completion of the original image stack. It is worth noting that similar regularized matrix factorization algorithms have been proven effective in solving complex real-world matrix completion problems^37, 38^, e.g., the Netflix Prize.

Our sparse spectroscopic stimulated Raman scattering (SS-SRS) imaging technique benefits from several innovations. First, we developed a uniform pseudo-random scanning scheme by designing a three-dimensional (3D) triangular Lissajous trajectory with a high least common multiplier (LCM) for the axis frequencies. Second, we designed a sparse spectroscopic image unmixing algorithm, which introduces regularizations based on the generalized Gaussian Markov random field (GGMRF)^39^, to make the solutions match the properties of natural images and spectra. Third, by tuning the beat frequency (*ω*_p_−*ω*_s_) through an off-axis galvo-mirror^22^, we experimentally implemented SS-SRS by scanning three galvo mirrors to guide the scanning path along the designed Lissajous trajectories. Using this framework, we successfully recovered spectral profiles and concentration maps from ~20% of pixels measured in a spectroscopic image stack. Consequently, at a pixel dwell time of 2 μs, we acquired a 50-frame spectroscopic SRS stack within 0.8 s. Such speed allows high-fidelity imaging of chemical compositions in highly dynamic fungal cells.

## Materials and methods

### Determination of the uniform random sampling pattern

**Figure 1:**
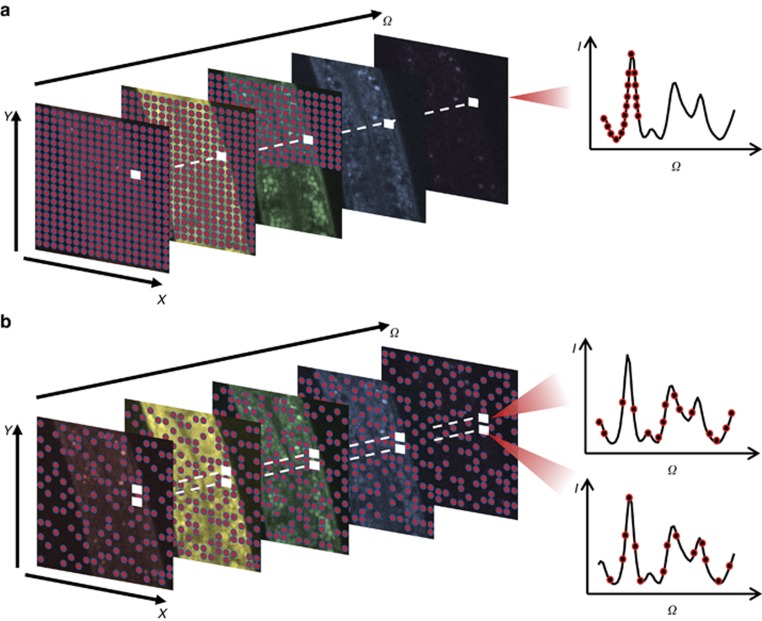
Comparison of data acquisition schemes for scanning spectroscopic imaging. (**a**) Conventional frame-by-frame raster-scanning design for a laser-scanning spectroscopic imaging system. (**b**) Sparse sampling by random collection of measurements throughout the entire spectroscopic image stack. The random sampling scheme fulfills the theoretical requirement for accurate reconstruction of a sub-sampled low-rank matrix (i.e., spectroscopic image).

As indicated by the literature on matrix completion, the sampled entries in the data matrix must be spread out randomly to avoid information loss^32^. In the context of scanning systems, the comparison between conventional scanning and random scanning is illustrated in Figure 1. Figure 1a shows a typical frame-by-frame design in which a complete image is scanned in raster order at each excitation wavelength to generate a spectroscopic image stack. As a comparison, Figure 1b shows a sparse sampling scheme in which a small portion of pixels is randomly and uniformly measured. The under-sampled image stack is subsequently used to computationally recover the complete image.

**Figure 2:**
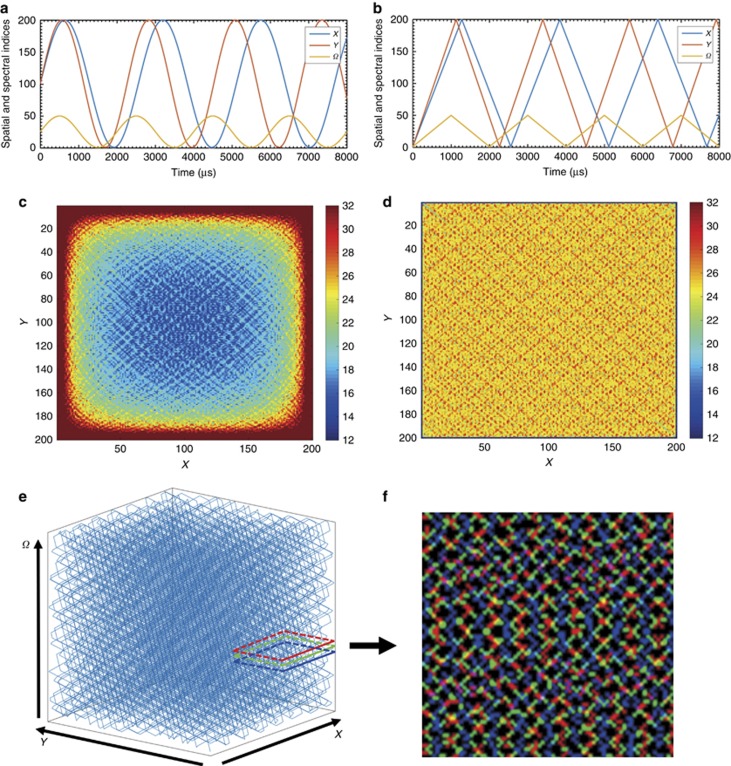
Practical sparse sampling design for spectroscopic SRS via the Lissajous trajectory. The 3D Lissajous trajectory with high least common multipliers for the scanning frequencies can generate a complicated pattern to achieve pseudo-random sampling. Panels (**a**) and (**b**) demonstrate the output waveforms of three axes by sinusoidal waves and triangular waves, and the corresponding spatial distributions of the one million measurements is shown in panels (**c**) and (**d**), respectively. Comparison of the two designs suggests that the triangular wave is advantageous because it supplies a more balanced sampling density. By dividing the non-repeating trajectory into sub-trajectories per the fill rate, each sub-trajectory forms a sparsely sampled spectroscopic image stack, panel (**e**) illustrates the 3D view of one sparsely sampled spectroscopic image with a 15% fill rate. (**f**) Projection of sampled pixels in a small spatial region from three consecutive frames (labeled as red, green and blue) shows that, at each spectral frame, the 3D trajectory can sample a subset of pixels that scatter throughout the entire scene and largely differs from frame to frame.

For SRS, the pixel dwell time is a few microseconds, which leaves a notably short time interval between two consecutive pixel measurements. The time constraint makes positioning and stabilizing the laser extremely challenging. Therefore, it is more practical to make a series of measurements that follow a continuous trajectory. To implement random sampling with the abovementioned constraints, we propose a 3D Lissajous trajectory, which can be generated by setting all three axes (*X*, *Y*, Ω) to follow replicating waveforms. Different from raster scanning, which moves to the next pixel by changing only one index among all of the axes, the Lissajous scanning trajectory changes all of the axis indices simultaneously. Such a pattern guarantees that, when the image is under-sampled, the measured pixels are still scattered across the entire image stack. Moreover, the trajectory is practical in that its complexity positively correlates with the LCM of the axis frequencies. In other words, a high-LCM Lissajous trajectory follows a complicated pattern which, given sufficient time, covers nearly all of the pixels in the stack and never repeats itself. Several two-dimensional sampling designs based on sinusoidal Lissajous trajectories have been reported for applications, such as two-photon fluorescence excitation microscopy^40, 41^, atomic force microscopy^42, 43^ and second harmonic generation microscopy^44^. However, the sinusoidal wave fails to create a uniform sampling pattern. Figure 2a and 2b, depicts the time series waveforms of the *X*, *Y* and Ω axes for two different designs, namely, sinusoidal and triangular. By mapping the 3D location of the sample at each time point, a trajectory is generated. After sampling 1 million pixels, the spatial distribution of the samples for the sinusoidal wave Lissajous trajectory is illustrated in Figure 2c. Clearly, more samples are located at the edge of the image, which consequently leads to an undesirable biased image reconstruction quality with a lower quality towards the center. In comparison, the triangular wave design supplies a much more uniform sampling density (Figure 2d), thus alleviating the problem. Based on this analysis, we selected the 3D triangular Lissajous trajectory to generate random measurements.

To generate a stream of under-sampled spectroscopic image stacks, the continuous yet complex trajectory is divided into a time series of sub-trajectories, each of which constitutes an individual stack. Thus each stack contains a random portion of the sampled pixels taken along the sub-trajectories. The length of each sub-trajectory determines the number of sampled pixels in the stack. We define the ratio of the sampled pixels over the total number of pixels as the fill rate, which indicates the achievable speed increase (the inverse of fill rate). A sub-trajectory with 15% fill rate is illustrated as a 3D view of the stack in Figure 2e. To show that our proposed sampling trajectory takes uniform and random samples in each spectral frame, we crop a small spatial region, project the sampled pixels in three adjacent spectral frames and differentiate them using different colors (Figure 2f). Consequently, limited sampled pixels spread out in the stack to supply sufficient information for reconstruction.

### Regularized sparse spectroscopic image unmixing

We adopted a regularized matrix factorization algorithm to reconstruct the image from raw measurements. First, we define *N*_*x*_, *N*_*y*_ and *N*_*λ*_ as the dimensions of the spectroscopic image, *N*=*N*_*x*_*N*_*y*_*N*_*λ*_ as the total number of pixels and *M* as the number of sampled pixels. Treating 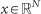
as the true image and 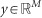
as incomplete and noisy measurements, the relationship between *x* and *y* can be expressed as follows:


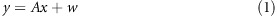


where 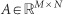 represents the modulation matrix describing how each measurement in *y* is mapped to *x*, and 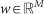 is assumed to be additive Gaussian noise. In addition, we use a linear mixing model to represent the spectroscopic image as a linear combination of spectral signatures. Assuming that *K* spectral signatures exist, the complete spectroscopic image can be described as follows:


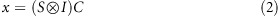


where 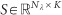 is the matrix containing the spectra of all spectral signatures. We arrange 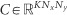 as a column vector that stores the concentrations of all spectral signatures in raster order. In addition, *I* is the identity matrix of size 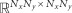, ⊗ represents the Kronecker product and thus 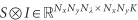. Combining Equations (1) and (2), the imaging process is formulated as follows:


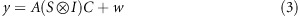


The developed algorithm aims to solve the inverse of Equation (3) to reconstruct *S* and *C*. Because the inverse problem is not jointly convex for *S* and *C*, we select an alternating optimization approach that iteratively optimizes one variable while holding another fixed.

With the alternating optimization approach, we first fix *S* and subsequently derive a maximum *a posteriori* (MAP) estimation for *C*. Let *H*≡*A*(*S*⊗*I*) be a modified modulation matrix when *S* is fixed, and thus Equation (3) is transformed into *y*=*HC*+*w*. Modeling the additive noise as an independent and identically distributed zero-mean Gaussian distribution with variance 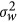, we can formulate the forward model *P*(*y*|*C*) as follows:


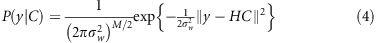


The prior model *P*(*C*) is used to describe the prior knowledge of *C*, which is a flexible approach to introducing regularizations. We split *C* into *K* channels such that 
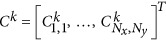
 for all 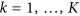. A GGMRF^39^ model is used to correlate neighboring pixel values while maintaining the sharp edges of an image:


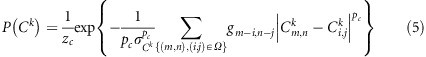


where *z*_*c*_ is a normalizing constant, 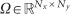 is the set of spatial indices, 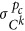 is the hyper-parameter, *g*_*m−i,n−j*_ represents the neighborhood weight and *p*_*c*_∈(1,2) is a predefined constant used to control the level of penalty for adjacent intensity change. Combining Equations (4) and (5), we can derive a convex optimization problem with respect to *C* that achieves the optimal estimate 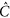:


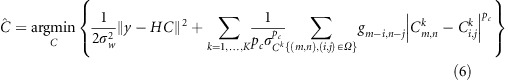


subject to the non-negativity constraint.

Similarly, we derive a MAP-GGMRF framework for *S* while holding *C* fixed. For notation simplicity, we reshape the spectral signatures *S* into a column vector 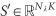, and concentration maps *C* into a matrix 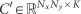 such that each row in 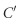 includes concentrations of all *K* spectral signatures at a particular spatial location. Defining 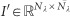, we formulate another modulation matrix 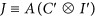, and subsequently, the original Equation (3) is rewritten as 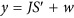. Based on the modified equation, the forward model for 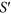 is given by the following:


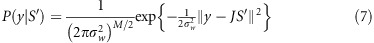


We use a prior model to phrase the assumption that, for each spectral signature, the spectral signature is more likely to smoothly change shape. For each of the *K* channels, the prior model is given by the following:


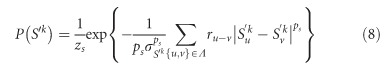


where *z*_*s*_ is the normalizing constant, 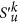 represents the value of *k*^th^ spectral signature at channel *u*, Λ is the set of spectral channels, 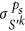 is the hyper-parameter for the spectral prior model, *p*_*s*_∈(1,2) is a constant with the same effect as *p*_*c*_ and *r*_*u−v*_ specifies spectral neighborhood weights. Combining Equations (7) and (8) yields the convex optimization problem, which solves the optimal estimate 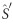:


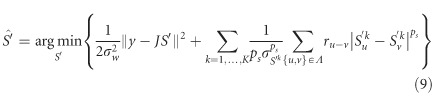


Given an initialization to one of the variables, the joint optimization is achieved by solving Equations (6) and (9) in an alternating and iterative manner. An iterative coordinate descent optimization^45, 46^ is used to solve the equations in a pixel-by-pixel manner (see Supplementary Information).

### Instrumentation strategy

**Figure 3:**
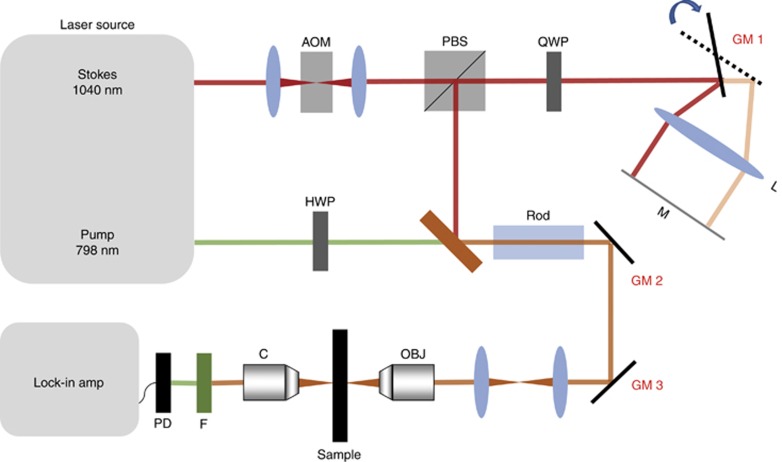
Optical setup for SS-SRS. Spectroscopic SRS imaging is achieved by tuning the temporal delay of the chirped Stokes beam and pump beam. A glass rod is applied for the chirping pulses. Temporal delay is added by directing the Stokes beam at the edge of GM1. Together with GM2 and GM3 for spatial positioning of the combined laser beams, the 3D high-LCM triangular Lissajous scanning trajectory is generated. AOM, acousto-optic modulator; C, condenser; F, filter; HWP, half-wave plate; L, lens; M, mirror; OBJ, objective; PBS, polarizing beam splitter; PD, photodiode; QWP, quarter-wave plate.

Based on the analysis in previous sections, we chose the 3D triangular Lissajous trajectory as the optimal pattern of sparse sampling. Such a pattern requires three axes to follow triangular waveforms simultaneously with precisely controlled frequencies. The waveforms for spatial axes (*X*, *Y*) can be achieved by sending triangular waveforms to a pair of galvo mirrors for laser positioning (labeled as GM2 and GM3 in Figure 3). To generate a triangular waveform in the frequency domain (Ω), a stable and high-speed frequency tuning is required and was achieved using the home-built delay-line tuner (Figure 3) reported in our previous work^22^, which introduced the optical path difference for the Stokes beam using a galvo mirror (labeled as GM1 in Figure 3). In brief, the Stokes beam was directed to the edge of the galvo mirror. After reflection, the beam was focused by an achromatic lens to a flat mirror and was subsequently reflected to the same optical path. Consequently, the movement of the galvo mirror introduced an optical path difference of a few millimeters for the retroflected Stokes beam. The introduced path difference was used to tune the frequency difference after chirping both beams. Experimentally, the three waveforms at carefully selected frequencies were generated by a multifunction data acquisition card and sent to the three galvo mirrors, and thus a high-LCM triangular Lissajous trajectory was generated.

The set-up for SS-SRS is illustrated in Figure 3. A tunable laser generates two synchronized outputs as the pump and Stokes beams at a repetition rate of 80 MHz. The Stokes beam was fixed at a 1040 nm wavelength, and the pump beam was tunable from 680 to 1300 nm. With modulation by an acousto-optic modulator operating at 2.4 MHz, the Stokes beam was engineered to add a path difference via our home-built delay tuner by which the Stokes beam was directed to the edge of the galvo mirror. After reflection, the beam was focused by an achromatic lens to a flat mirror and was subsequently reflected to the same optical path. Consequently, the movement of the galvo mirror introduced an optical path difference of a few millimeters for the retroflected Stokes beam. The introduced path difference was used to tune the frequency difference after chirping both beams. Experimentally, the three waveforms at carefully selected frequencies were generated by a multifunction I/O card (PCIe 6363, National Instrument) and sent to the three galvo mirrors, and thus a high-LCM triangular Lissajous trajectory was generated.

The combined use of a quarter-wave plate and a polarizing beam splitter separated out the retroflected Stokes beam. The pump beam first passed through a half-wave plate to match the polarization of the Stokes beam. The two beams were combined and chirped by glass rods to stretch in the time domain and focus the energy in the spectral domain. Eventually, the chirped pulses were sent to a laser-scanning microscope. A 60 × objective (UPLSAPO 60XW, Olympus) was used to focus the laser on the sample. The maximum laser powers were 12 mW for 800 nm and 80 mW for 1040 nm. The output SRS signals were filtered and collected by a photodiode (S3994, Hamamatsu), which was incorporated using a home-built resonant circuit with a central frequency of 2.4 MHz with a 300-kHz bandwidth. The collected data were sent to a digital lock-in amplifier with a time constant of 1 μs (HF2LI, Zurich Instrument). Finally, a digitizer (PCI 6110, National Instrument) was used to record the data at a 1.5 MHz sampling rate.

### Preparation of microsphere mixture

A mixture of polystyrene (PS) and poly(methyl methacrylate) (PMMA) beads (5 μm diameter, Phosphorex) was prepared by the mixing microsphere solutions together with water. A droplet of solution was placed on a coverslip and sealed for imaging.

### Preparation of live fungal cells

*Candida albicans* isolates (strain number 55) were cultured in yeast extract peptone dextrose overnight at 37 °C with 250 r.p.m. shaking. The 500 μl Candida suspension was centrifuged, washed three times with phosphate-buffered saline and diluted in phosphate-buffered saline. A droplet of solution with diluted Candida was placed on a coverslip and sealed for imaging.

## Results and discussion

### SS-SRS imaging of microbeads in Brownian motion

**Figure 4:**
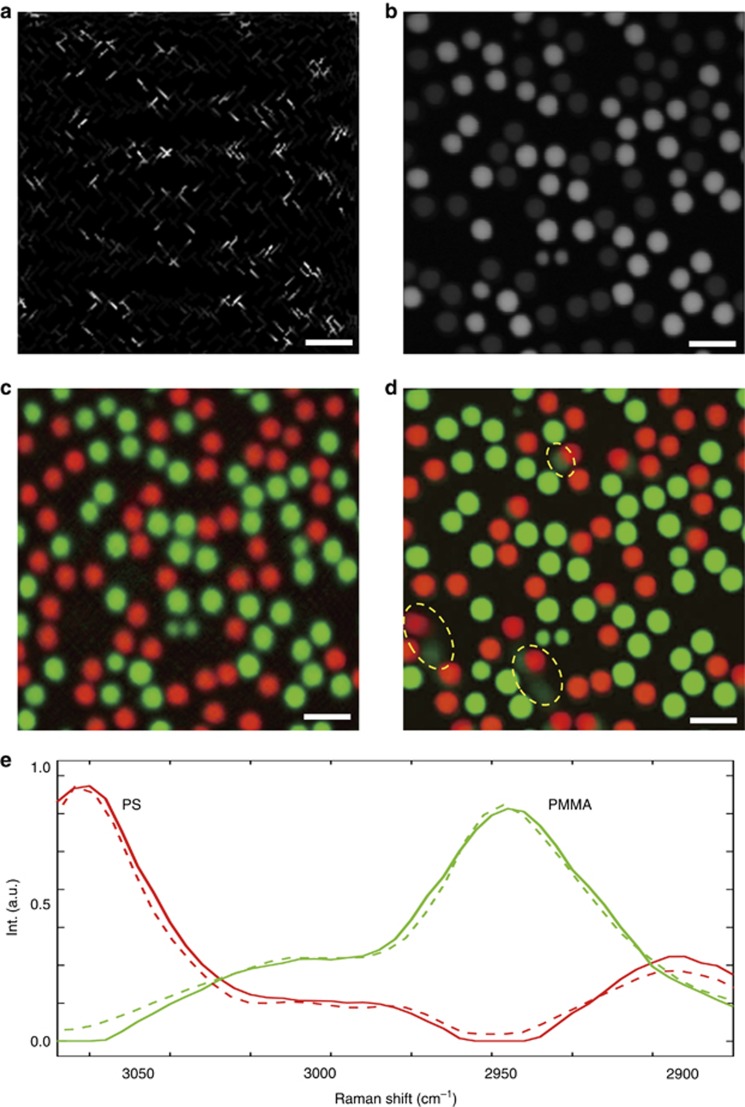
Experimental results for PS and PMMA microbead mixture in water. (**a**) One frame of the sparsely sampled raw spectroscopic image at 2915 cm^−1^ with a pixel dwell time of 2 μs; the entire spectroscopic SRS data cube with 50 frames was captured in 0.8 s. (**b**) One frame of the raster-scanned spectroscopic SRS image at 2915 cm^−1^; the stack was captured at a speed of 2 frames s^−1^. Output concentration maps using regularized spectroscopic image unmixing for the (**c**) sparsely sampled image and (**d**) raster-scanned image, respectively. Motion artifacts induced by high bead motility are shown in **d** (selected examples are highlighted by yellow circles). (**e**) Output spectral signatures for sparsely sampled image (solid line) and raster-scanned image (dotted line). Scale bars, 10 μm.

To demonstrate the advantage of SS-SRS over a conventional frame-by-frame SRS system, we imaged 5-μm diameter PS and PMMA beads mixed in water at a pixel dwell time of 2 μs. Using the proposed sparse sampling trajectory, we generated 200 × 200 × 50 SS-SRS image stacks with a 2 μs pixel dwell time. Approximately 20% of pixels were sampled, resulting in an acquisition speed of 0.8 s per stack. Figure 4a illustrates one spectral frame at 2915 cm^−1^, from which the randomness of sampling in spatial dimension is confirmed. In addition, a frame-by-frame view of the entire raw image stack is shown in Supplementary Video 1 to further demonstrate the randomness of sampling in a 3D sense. After applying the unmixing algorithm to the raw image, both the spectral signatures and concentration maps are generated. For comparison, we recorded a 50-frame raster-scanned spectroscopic image stack at a speed of 2 frames s^−1^. One spectral frame taken at 2915 cm^−1^ is shown in Figure 4b, and a frame-by-frame view of the stack is presented in Supplementary Video 2. The video clearly indicates that certain beads changed positions drastically from one spectral frame to another, showing high motility due to the Brownian motion in second scale. The stacks were decomposed as spectral signatures and concentration maps using the same unmixing algorithm. The spectral signatures output using the sparsely sampled image (solid line) and raster-scanned image (dotted line) are depicted in Figure 4e. The concentration map (Figure 4c) for the sparse image is free of motion artifacts and maintains desirable image resolution from which we can clearly distinguish the morphology and spatial distributions of two types of beads. In contrast, the output concentration maps for the raster-scanned spectroscopic image (Figure 4d) show significant motion artifacts (circled in yellow). To further demonstrate the speed advantage of SS-SRS system, we recorded 6 consecutive spectroscopic stacks at a speed of 0.8 s per stack, and the video of concentration maps generated from the stream of reconstructed image stacks can be found in Supplementary Video 3, which demonstrates the sample dynamics with a desirable spectral and spatial resolution. In the above analysis, we selected a 20% fill rate because a higher fill rate did little to improve the quality. We also tested lower fill rates and found that PS and PMMA particles can be well separated at less than a 5% fill rate (See Supplementary Fig. S1). Under such condition, we were able to acquire the entire stack within 0.16 s.

It is important to compare the spatial resolution of the sparsely sampled image with that of raster-scanned images. Comparison between Figure 4c and 4d, shows that, although we reduced the number of sampled pixels by 5 times, the spatial resolution decreased only by ~1.5 times, from 758 nm to 1.12 μm (detailed quantification of spatial resolution can be found in Supplementary Information). The spatial resolution is effectively maintained because sampling in the spectroscopic domain is used in reconstruction of the final chemical maps. The slight decrease is likely caused by the GGMRF prior model, which stabilizes the output when certain pixels lack information by making the neighboring pixels follow Gaussian-like distributions with similar means. In the future, the resolution in the sparsely sampled images can be further improved from a hardware aspect by optimizing the sampling trajectory using polygon scanners and from an algorithm aspect by applying more advanced prior models that consider non-local neighborhood information (e.g., non-local means^47^, block-matching 3D filtering^48^).

### SS-SRS imaging of living fungal cells

**Figure 5:**
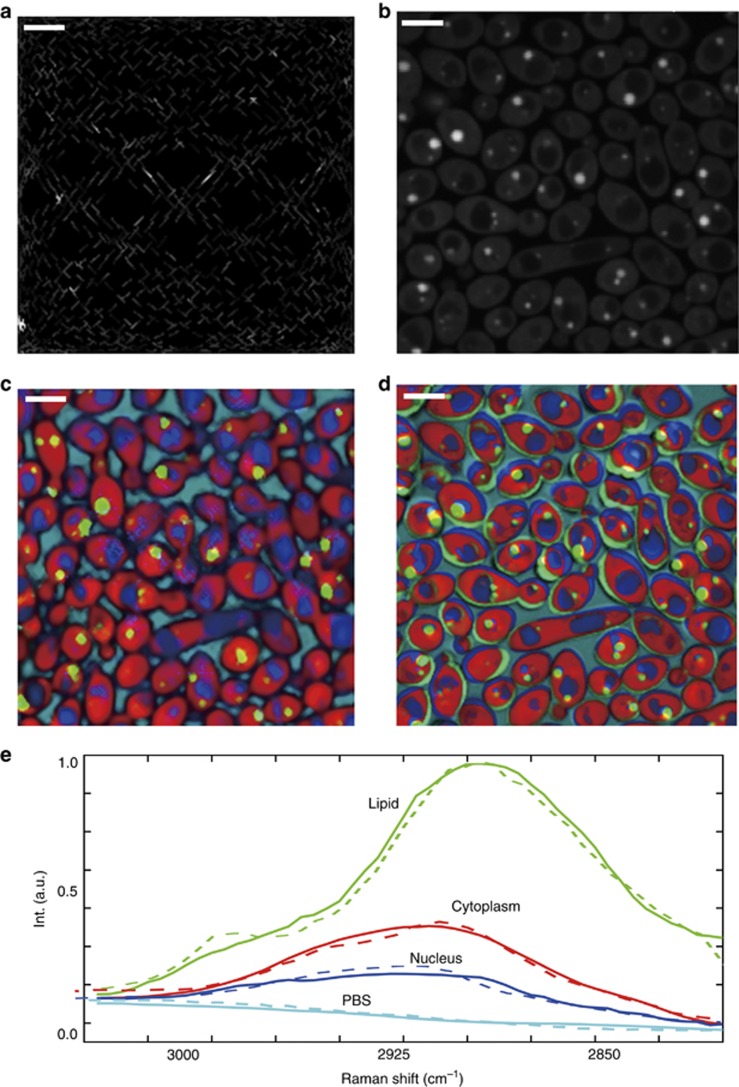
Experimental results for living *C. albicans* in PBS buffer. (**a**) One frame of the sparsely sampled raw spectroscopic image at 2920 cm^−1^ with a pixel dwell time of 2 μs; the entire spectroscopic SRS data cube with 50 frames was captured in 0.8 s. (**b**) One frame of a raster-scanned spectroscopic SRS image at 2920 cm^−1^; the stack was captured at a speed of 2 frames s^−1^. Output concentration maps using regularized spectroscopic image unmixing for (**c**) sparsely sampled image and (**d**) raster-scanned image, respectively. Motion artifacts in **d** severely distort the spatial distributions and the concentrations of multiple components. (**e**) Output spectral signatures for sparsely sampled image (solid line) and raster-scanned image (dotted line). Scale bars, 10 μm.

Our imaging system can resolve chemical compositions in a more complex environment, such as highly dynamic living cells, to enable fast detection of pathogens (e.g., fungi or bacteria) in natural environments. To demonstrate the performance, we used the same configuration to perform SS-SRS imaging of fungal cells *C. albicans* in a growth medium. Compared with beads, the irregular shape of the cells and the small lipid droplets within the cells imposed additional challenges. Using the same fill rate as in the previous experiment, we acquired an SS-SRS spectroscopic image stack at a speed of 0.8 s per stack. Figure 5a shows one frame of the sparsely sampled raw image at 2920 cm^−1^. A frame-by-frame view of the stack can be found in Supplementary Video 4. In comparison, a raster-scanned frame-by-frame spectroscopic image stack was taken at a speed of 2 frames s^−1^ (see Figure 5b for one frame taken at the same excitation wavelength and Supplementary Video 5 for a frame-by-frame view). Similar to the previous bead imaging case, we observed from Supplementary Video 4 that the cell motility was significant from frame to frame. By running the singular value decomposition and evaluating the number of significant eigenvalues, we identified four major components from the image stack. After applying the unmixing algorithm to both the sparse data and the reference data, we generated the corresponding concentration maps of the nucleus, cytoplasm, lipids and medium (Figure 5c and 5d). Figure 5c indicates that most of the small lipid droplets were clearly captured using our platform. In comparison, selected lipid droplets in Figure 5d are located outside the cell body and are colored incorrectly due to cell motility. Figure 5e shows the output spectral signatures of the nucleus, lipid, cytoplasm and medium for both the sparsely sampled image (solid line) and raster-scanned image (dotted line), which confirms that our system could resolve the four components in the sparsely sampled condition. A video of the concentration maps for 6 consecutive spectroscopic image stacks was recorded within 4.8 s (Supplementary Video 6) in which we can observe the motion of *C. albicans*.

The above experimental results show that, under a pixel dwell time of 2 μs and a sampling fill rate of ~20%, a 200 × 200 × 50 spectroscopic image stack can be recorded within 0.8 s. This speed was achieved without sacrificing pixel dwell time by drastically reducing the sampled pixels in the spectroscopic image. Currently, the speed is limited by the low speed (~1 kHz) of the galvo mirrors. Under such a speed, the mirrors could not closely follow triangular waves at the crests and troughs, rendering the sampling less uniform than expected. To overcome this limitation, the galvo mirror could be replaced with a polygon scanner in a future project. Because a polygonal scanner can produce a continuous linear sawtooth scanning pattern by rotating the mirror facets at notably high speeds, the generated sampling pattern is expected to be more uniform. By driving the pixel dwell time to 0.1 μs, which has been proven feasible in video-rate single-color SRS imaging^13^, it is expected that SS-SRS can ultimately push the speed limit of spectroscopic SRS imaging to video rate.

For reconstruction of a single spectroscopic image, the Matlab version of the algorithm required approximately 1 min to complete on a personal laptop with an Intel i7-4700MQ CPU. As previously proved, the algorithm can be implemented in parallel without affecting the results if an appropriate surrogate function is applied to the original GGMRF model^49^. Thus the speed of the algorithm can be greatly enhanced if the program is written in a parallel manner and run on GPUs or clusters of CPUs.

## Conclusions

We reported a sparsely sampled SRS system that could capture a high-resolution spectroscopic image stack covering a 200 cm^−1^ window within 0.8 s. Such a distinct advantage in speed and resolution enables resolution of chemical components in a highly dynamic environment. Real-time imaging of freely moving microbeads and fungi were demonstrated, proving that our system enables the use of spectroscopic Raman imaging for real-time detection of microorganisms and chemical mapping of organelles in living cells and tissues. Although not demonstrated in this paper, the trajectory design has the potential for coupling to a variety of high-dimensional laser-scanning imaging modalities.

## Author contributions

HL, C-SL and PW designed the sampling trajectory; HL and NK designed and implemented simulations; C-SL built the system; HL and C-SL performed experiments; HL coded the reconstruction program and analyzed the data; HL, C-SL, NK and J-XC wrote the manuscript; and NK and J-XC supplied overall guidance for the project.
