# Supplementary material for: Spectroscopic stimulated Raman scattering imaging of highly dynamic specimens through matrix completion
Source: Light Sci Appl. 2018 May 4;7:17179–. doi: 10.1038/lsa.2017.179 (PMC6060072; doi:10.1038/lsa.2017.179)
Supplement: Supplementary Information [file lsa2017179x1.doc]

**Supplementary information**

**Spectroscopic Stimulated Raman Scattering Imaging of Highly Dynamic Specimens through Matrix Completion**

Haonan Lin[1](http://staging-www.nature.com/lsa/journal/v7/n4/full/lsa2017179a.html#aff1),[*](http://staging-www.nature.com/lsa/journal/v7/n4/full/lsa2017179a.html#note1), Chien-Sheng Liao[2](http://staging-www.nature.com/lsa/journal/v7/n4/full/lsa2017179a.html#aff2),[*](http://staging-www.nature.com/lsa/journal/v7/n4/full/lsa2017179a.html#note1), Pu Wang[3](http://staging-www.nature.com/lsa/journal/v7/n4/full/lsa2017179a.html#aff3), Nan Kong[4](http://staging-www.nature.com/lsa/journal/v7/n4/full/lsa2017179a.html#aff4) and Ji-Xin Cheng[1](http://staging-www.nature.com/lsa/journal/v7/n4/full/lsa2017179a.html#aff1),[2](http://staging-www.nature.com/lsa/journal/v7/n4/full/lsa2017179a.html#aff2)

1. Department of Biomedical Engineering, Boston University, Boston, MA 02215, USA
2. Department of Electrical & Computer Engineering, Boston University, Boston, MA 02215, USA
3. Vibronix, Inc., West Lafayette, IN 47907, USA
4. Weldon School of Biomedical Engineering, Purdue University, West Lafayette, IN 47907, USA

^*^These authors contributed equally to this work.

**Solving the cost functions**

Due to the high dimensionality, equations (6) and (9) are numerically challenging to solve using gradient descent algorithm. Here we use an iterative coordinate descent (ICD) algorithm^1,2^, which minimizes the cost functions with respect to one coordinate each time while keeping the rest fixed, a complete iteration thus includes sequential updates for all coordinates once.

First, we focus on the optimization of equation (8). Defining an error vector $e-y-HC$, the ICD update for a pixel $C_{m,n}^{k}$ can be written as follows:

|  | $\beta\leftarrow C_{m,n}^{k}$,  $l\leftarrow(m\times N_{x}+n-1)\times K+k$,  ,  $e\leftarrow e-H_{*,l}(C_{m,n}^{k}-\beta)$. | (S) |
| --- | --- | --- |

Where $H_{*,l}$represents a vector containing all elements in column $l$, and $\partial C_{m,n}^{k}$indicates a neighborhood set for pixel (m,n). In this work, we select $\partial C_{m,n}^{k}$ as an 8-neighborhood system with weights either 1/12 or 1/6, depending on the distance to the central pixel. The cost function in equation (S1) contains a non-quadratic term with an order of $p_{c}\in(1,2)$, which imposes challenge in finding its minimum value efficiently. To address the issue, we apply a surrogate function to the original GGMRF prior model, such that the non-quadratic term is transformed to a quadratic approximate. This technique, termed q-GGMRF, has been successfully applied to solving computed tomography (CT) reconstruction problems^3^. Subsequently, the solution at each pixel could be easily solved by setting the first-order derivative to zero. A specific type of surrogate function, termed symmetric bound^4^, is used to adjust the original spatial neighborhood weight:

|  | $\tilde{g}_{m-i,n-j}\leftarrow g_{m-i,n-j}\frac{\left\vert C_{i,j}-C_{m,n}\right\vert^{p_{c}-2}}{2\sigma_{C^{k}}^{p_{c}}}\frac{\frac{\left\vert C_{i,j}-C_{m,n}\right\vert^{2-p_{c}}}{T\sigma_{C^{k}}}\left(\frac{2}{p_{c}}+\left\vert\frac{\left\vert C_{i,j}-C_{m,n}\right\vert^{2-p_{c}}}{T\sigma_{C^{k}}}\right\vert\right)}{\left(1+\frac{\left\vert C_{i,j}-C_{m,n}\right\vert^{2-p_{c}}}{T\sigma_{C^{k}}}\right)^{2}}$. | (S) |
| --- | --- | --- |

The original ICD update in equation (S1) can then be transformed as the following:

|  | . | (S) |
| --- | --- | --- |

Using the same method, we can derive the ICD update for the spectral profiles. The pseudocode for the algorithm is presented below:

**Pseudocode for sparse spectroscopic algorithm implementation using surrogate for prior model**


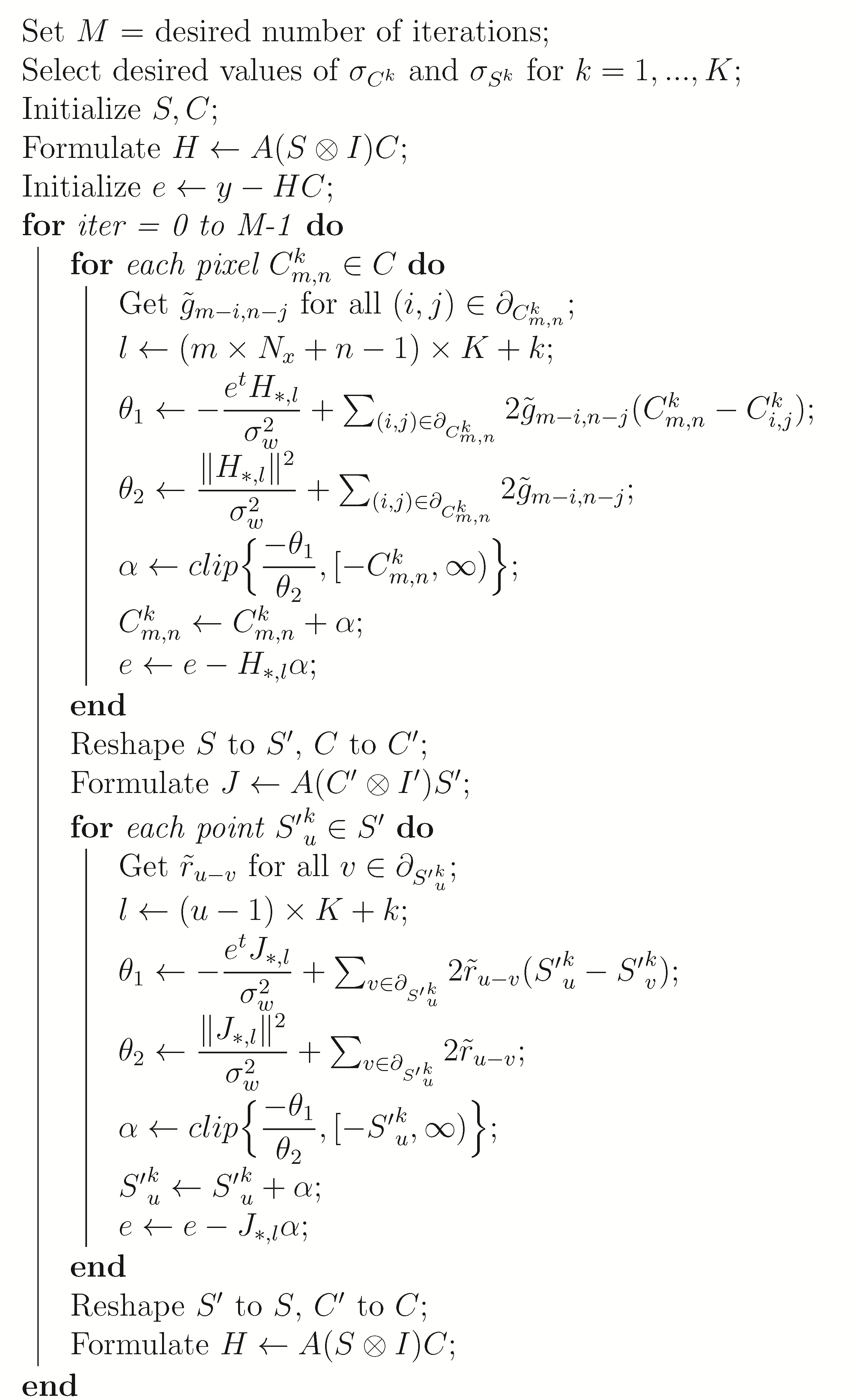


**Initialization for S and C**

To obtain satisfying unmixing results, it is important to give an appropriate initialization on C and S. In this work, we develop an initialization procedure based on the properties of sparse spectroscopic images.

First, we give initial guess on spectral profiles. Based on unique shape information of spectroscopic images, a projection of the raw sparse image stack along the spectral dimension is taken to form a 2D image, which provides a rough spatial information of the imaging objects. Based on the projection image, segmentation of the regions representing the same substance is performed either manually or using super-pixel analysis such as simple linear iterative clustering (SLIC)^5^. Each segmented spatial region then outputs an averaged spectral profile by taking the mean of sampled values at each spectral channel. After extracting a set of spectral profiles, we determine the number of pure components by running singular value decomposition (SVD) and evaluating the number of significant eigenvalues^6^. Finally, initial guess on spectral signatures is performed by running automated endmember extraction algorithms (e.g., pixel purity index^7^, evolving factor analysis^8^, etc.).

The initialization of concentration map C is performed by solving for C as the least square solution of equation (3) provided S is fixed as the initial guess. If the data has very low fill rate, or the spectral profiles are not distinctly different, the least square solution becomes undesirable. To overcome this problem, we apply $l_{1}$-norm regularization to drive insignificant components towards zero. Non-background pixels that are driven to zero can be later restored by the proposed unmixing algorithm.

**Hyper-parameter selection**

Hyper-parameters, $\sigma_{w}$,$\sigma_{C^{k}}$ and, control the level of regularization. As the level increases, the solution is more significantly contributed by its neighbors, and the image becomes oversmoothed. On the other hand, if the level decreases, the solution is mostly contributed by the forward model term, which is undesirable when the data is noisy and sparse. Here, we elect to take the maximum-likelihood (ML) estimate for estimating the hyper-parameters. Setting the first-order derivative of equation (5) to zero and solving for $\sigma_{C^{k}}$, we can derive the ML estimate for the concentration map parameters:

|  | $\sigma_{C^{k}}^{p_{c}}=\frac{1}{N_{x}N_{y}}\sum_{\{(m,n),(i,j)\in\Omega\}} g_{m-i,n-j}\vert C_{m,n}^{k}-C_{i,j}^{k}\vert^{p_{c}}\}$. | (S) |
| --- | --- | --- |

Likewise, using equation (8), we have:

|  | . | (S) |
| --- | --- | --- |

For the forward model parameter $\sigma_{w}$, equation (3) can be used after S and C are initialized. Define M as the total number of sampled entries in the original sparse image, $\Gamma$ as the collection of sampled entries, the estimate for forward model parameter is as follows:

|  | $\sigma_{w}^{2}=\frac{1}{M}\sum_{i\in\Gamma} \left(y_{i}-\left[A(S\otimes I)C\right]_{i}\right)^{2}$. | () |
| --- | --- | --- |

**Quantification of spatial resolution**

As shown in **Figs. 4c** and **4d**, performing sparse sampling downgrades the spatial resolution. To quantify the difference, we measured intensity profile across the diameter of the 5-μm bead, and took first-order derivative of the profile. Two Gaussian-shape peaks (one positive and one negative) appeared at the edge and can be considered roughly as the point spread function of the system. The distance between the positive and negative peak corresponded to the diameter of the bead (5 μm), and the full-width at half maximum (FWHM) of the Gaussian peaks was calculated to quantify the blurring effect by performing sparse scanning. Comparison shows that, although we reduced the number of sampled pixels 5 times, the spatial resolution decreased by ~1.5 times, from 758 nm to 1.12 μm. The resolution change is contributed by multiple factors, depending on the number of components, the sampling trajectory pattern and the level of regularizations of the GGMRF prior model.


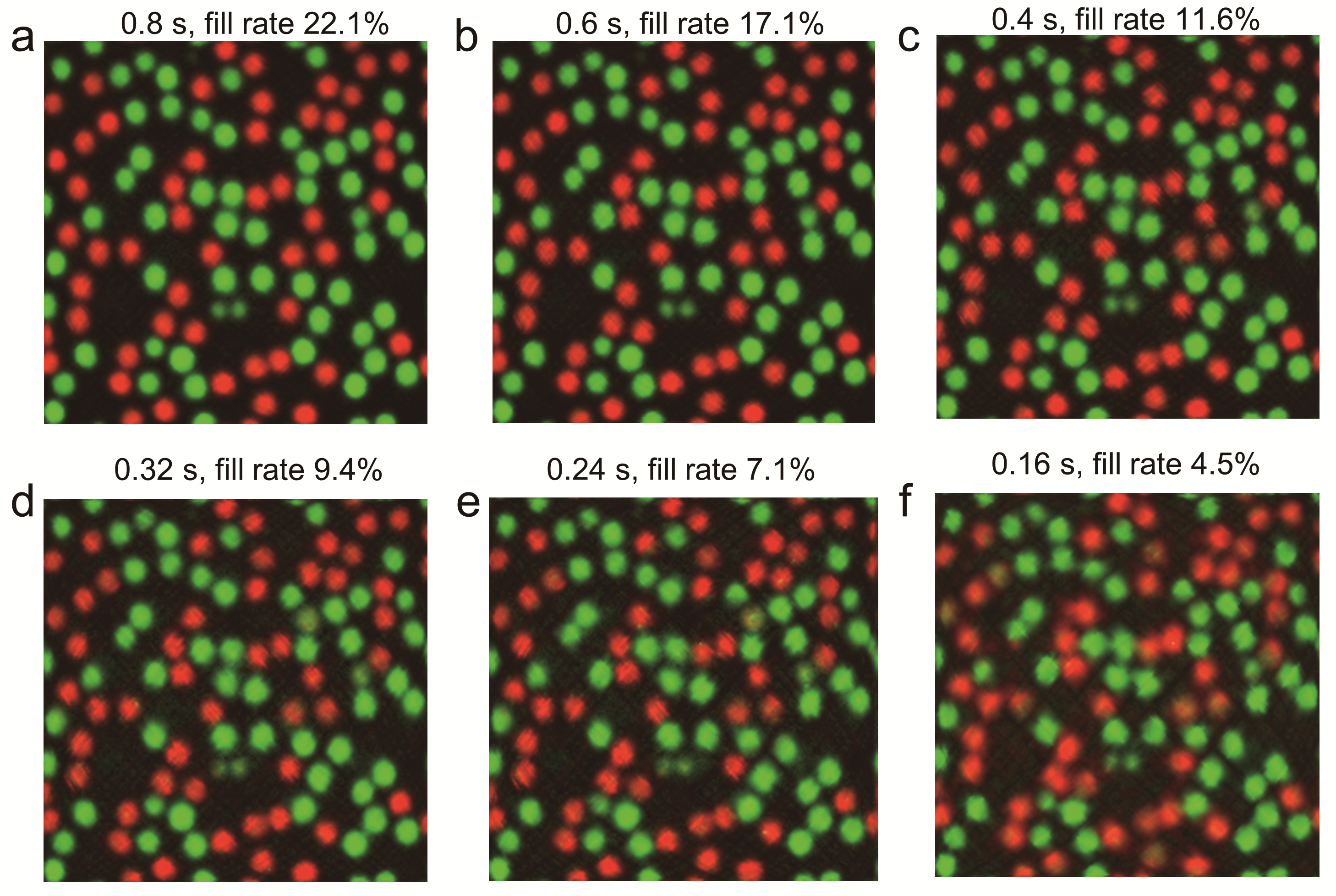


**Supplementary Figure S1. Output concentration maps using different sampling fill rates**. (a-f) Concentration maps using the sparse spectroscopic images of PS and PMMA with fill rates from 22.1% to 4.8%. As the fill rate decreases, the output beads images get distorted by the trajectory and the background increases. ~20% was used in the remainder of the experiments to maintain a good image quality.

**Supplementary Video Captions**

**Supplementary Video 1.** For PMMA and PS beads mixture, the frame-by-frame display of the sparsely sampled spectroscopic image stack. Scale bars, 10 μm.

**Supplementary Video 2.** Frame-by-frame raster-scanned spectroscopic SRS image stack of PS and PMMA. Acquisition speed, 2 frames/s. Between two consecutive frames, some beads showed significant motion, leading to severe motion artifacts (as shown in Fig 4(f)). Scale bar, 10 μm.

**Supplementary Video 3.** 6 consecutive stacks of concentration maps for PS+PMMA beads mixture in water. Beads motion were clearly observed due to Brownian motion. Scale bar, 10 μm.

**Supplementary Video 4.** For *C. albicans*, the frame-by-frame display of the sparsely sampled spectroscopic image stack. Scale bars, 10 μm.

**Supplementary Video 5.** Frame-by-frame raster-scanned spectroscopic SRS image stack of *C. albicans*. Acquisition time, 2 frames/s. Between two consecutive frames, significant sample motility was shown, both in terms of the fungal cells body and the lipid droplets within the cells. Scale bars, 10 μm.

**Supplementary Video 6.** 6 consecutive stacks of concentration maps for *C. albicans* in PBS buffer. Scale bar, 10 μm.

**REFERENCES**

1 Wright SJ. Coordinate descent algorithms. *Math Program* 2015; **151**: 3–34.

2 Bouman C, Sauer K. A unified approach to statistical tomography using coordinate descent optimization. *IEEE Trans Image Process* 1996; **5**: 480–492.

3 Thibault JB, Sauer KD, Bouman C, Hsieh J. A three-dimensional statistical approach to improved image quality for multislice helical CT. *Med Phys* 2007; **34**: 4526–4544.

4 Bouman C. *Model Based Image Processing*. Purdue University: West Lafayette, 2013.

5 Achanta R, Shaji A, Smith K, Lucchi A, Fua P, Süsstrunk S. SLIC superpixels compared to state-of-the-art superpixel methods. *IEEE Trans Pattern Anal Mach Intell* 2012; **34**: 2274–2281.

6 Jaumot J, Juan A De, Tauler R. MCR-ALS GUI 2.0 : New features and applications. *Chemom Intell Lab Syst* 2015; **140**: 1–12.

7 Chaudhry F, Wu C, Liu W, Chang C. Pixel purity index-based algorithms for endmember extraction from hyperspectral imagery. *Recent Adv Hyperspectral Signal Image Process* 2006; **37**: 29–61.

8 Maeder M, Zuberbuehler AD. The resolution of overlapping chromatographic peaks by evolving factor analysis. *Anal Chim Acta* 1986; **181**: 287–291.
